# Retinal microvascular changes in systemic lupus erythematosus assessed by optical coherence tomography angiography

**DOI:** 10.1186/s40942-024-00617-6

**Published:** 2024-12-18

**Authors:** André Ferreira, Luísa Viveiros, Raquel Faria, Francisca Bragança, Ana Carolina Abreu, Daniela Santos, Maria João Furtado, Miguel Lume, Pedro Menéres, José P. Andrade

**Affiliations:** 1 Department of Ophthalmology, Unidade Local de Saúde de Santo António, Largo do Prof. Abel Salazar, Porto, 4099-001 Portugal; 2 Department of Biomedicine - Unit of Anatomy, Faculty of Medicine, University of Porto, Porto, Portugal; 3 Centre for Health Technology and Services Research, Health Research Network (CINTESIS@RISE), Faculty of Medicine, University of Porto, Porto, Portugal; 4 Serviço de Medicina Interna, Unidade Local de Saúde de Santo António, Porto, Portugal; 5 Unidade de Imunologia Clínica, Centro Hospitalar Universitário de Santo António, Porto, Portugal; 6 Unidade Multidisciplinar de Investigação Biomédica, Instituto de Ciências Biomédicas Abel Salazar, Universidade do Porto, Porto, Portugal; 7 Department of Ophthalmology, Instituto de Ciência Biomédicas Abel Salazar, Universidade do Porto, Porto, Portugal

**Keywords:** Systemic Lupus Erythematosus, Autoimmune disease, Optical coherence tomography angiography, Vascular network

## Abstract

**Purpose:**

To assess the retinal microvasculature of Systemic Lupus Erythematosus (SLE) patients using Optical Coherence Tomography Angiography (OCTA).

**Methods:**

Twenty adult SLE patients without disease activity and no ocular manifestations were recruited and cross-sectionally assessed. A demographically similar cohort of healthy subjects was used for comparison. The main outcome is vascular density (VD). As secondary outcomes, foveal avascular zone (FAZ) parameters, total vessel length (TVL), vessel length density (VLD), vessel diameter and tortuosity, branchpoint density (BD) and fractal dimension (FD) were evaluated.

**Results:**

Forty eyes of 40 women (20 SLE patients and 20 healthy subjects) were enrolled with a mean ± SD age of 36.7 ± 9.9 years. In both the superficial and deep capillary plexuses, SLE patients presented significantly lower VD and TVL values, mean vessel diameter and FD (all *p* < 0.005). No significant differences were found in FAZ values. A negative correlation was found between age and FAZ parameters and between age and TVL, VLD and FD in the superficial capillary plexus (all *p* < 0.05).

**Conclusions:**

This study supports the evidence of subclinical retinal microvascular changes in adult SLE patients, providing a more comprehensive characterization of vascular changes. We found vessel diameter and length and FD are decreased in SLE.

**Trial registration:**

NCT05863689, 2023-10-19.

## Key messages

**What is known:**

SLE patients present reduced macular VD without overt ocular involvement when compared to healthy subjects.

**What this paper adds:**

OCTA is able to detect subclinical microvascular changes that can be characterized beyond the quantification of vascular density. The retinal microvascular system of SLE patients is reduced in density, length, and diameter, and the fractal dimension is also lower compared to healthy subjects.

## Introduction

Systemic lupus erythematosus (SLE) is a chronic autoimmune disease predominantly affecting females of childbearing age [1, 2]. The vascular network of several organs might be involved as this condition can induce vasculitis, increase the atherosclerotic and thrombotic burden and impair vasoregulatory mechanisms [3, 4], demonstrating its complex and multifactorial pathophysiology. In up to one-third of patients, ocular manifestations precede the systemic disease [5] with lupus retinopathy being one of the most severe complications, affecting up to 29% of patients with active disease [6]. These facts highlight the relevance of assessing the retinal microvasculature before overt manifestations occur. Not only is posterior segment involvement enough to score for active disease in the SLE Disease Activity Index 2000 (SLEDAI-2 K) tool as it is associated with increased activity and end-organ damage, particularly the central nervous system (CNS) and kidney [6].

Optical Coherence Tomography Angiography (OCTA) is a non-invasive imaging modality that allows the assessment of ocular vasculature without dye injection by comparing the differences between sequential OCT scans. A recent systematic review with meta-analysis from our group revealed that SLE patients without overt ocular involvement present a reduction of macular vessel density in the superficial and deep capillary plexuses [7]. Despite those studies only assessed vessel density and foveal avascular zone (FAZ) parameters, other quantitative metrics can further characterize retinal microvascular network, including vessel length, diameter, tortuosity, branchpoint density and fractal dimension. OCTA Vascular Analyzer (OCTAVA) is a new open-source software that can provide the metrics mentioned above for the study of retinal vascular networks in an accurate and reproducible way [8].

Therefore, in this work, we sought to characterize the retinal microvasculature of SLE patients with inactive disease and no overt ocular involvement using OCTA, in order to understand further the ocular vascular impairment documented in the literature [7].

## Methods

This research protocol followed the tenets of the Declaration of Helsinki [9] and was approved by the Institutional Review Board (IRB) of Unidade Local de Saúde de Santo António (ULS-SA; IRB file number: 2022.156(127-DEFI/128-CE)). All participants provided informed consent before enrolment, and the study protocol is registered in the Clinicaltrials. gov platform with the number NCT05863689.

### Participants

Patients fulfilling the 2019 European League Against Rheumatism (EULAR) / American College of Rheumatology (ACR) classification criteria for SLE [10] were prospectively selected from the Autoimmune Disease clinic of ULS-SA. Only adults with a disease duration below ten years, a mean daily dose of hydroxychloroquine (HCQ) inferior to 5 mg/kg, cumulative HCQ dose below 1000 g, no disease activity (SLEDAI-2 K ≤ 4 in the last 12 months) and no documented (past or present) ocular involvement were enrolled. A demographically similar cohort of healthy volunteers served as comparison.

Exclusion ophthalmological criteria included significant lens opacities, any retinal disease, high refractive error, ocular hypertension or glaucoma, previous intraocular surgery, or trauma. Arterial hypertension, diabetes mellitus, kidney diseases or other documented microvascular diseases, secondary Sjögren syndrome, local or systemic inflammatory diseases (other than SLE), smoking and pregnancy were deemed as exclusion systemic criteria.

### Protocol and data collection

A complete ophthalmological examination was performed on all participants, including best-corrected distance visual acuity assessment, anterior and posterior segments slit-lamp examination, auto-refractometer, and Goldmann applanation tonometry. All patients were scanned with Spectral-Domain OCT (SD-OCT) using Spectralis HRA + OCT (Heidelberg Engineering, Heidelberg, Germany) centered on the fovea for an OCTA scan using 10°×10° scan protocol. Low-quality scans were excluded.

Both groups’ past medical history, medications and other relevant clinical data were retrieved from medical records. The eye with the higher quality of OCT images was selected as the study eye when both fulfilled the criteria. If both eyes had the same quality of OCT image, the study eye was chosen by flipping a coin.

### Imaging analysis

**Fig. 1:**
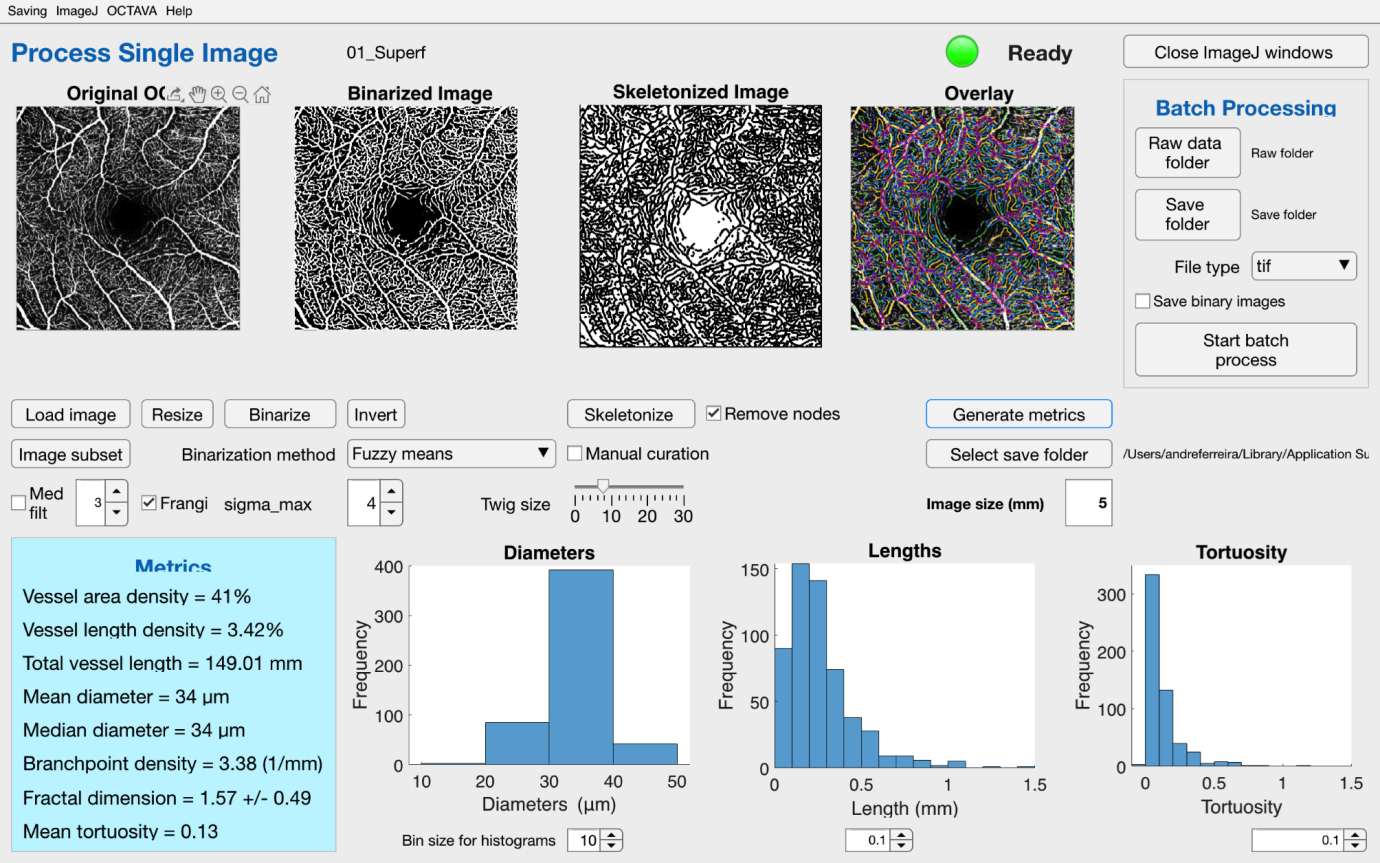
OCTAVA graphical user interface

Considering the OCTA scans analysis, automated segmentation of the superficial and deep capillary plexuses (SCP and DCP, respectively) was provided by Spectralis (Heidelberg Engineering) software (Version 6.14.1). The retinal projection artifacts were removed using the projection artifact removal tool. FAZ parameters were assessed in the superficial capillary plexus en-face angiogram using Image J 1.8.0 software (National Institutes of Health, Bethesda). FAZ perimeter (mm) and area (mm^2^) were manually outlined two times by a blind researcher as previously described [11] and the average of the two measurements was used. FAZ circularity was then calculated using the Eq. (4π × area)/perimeter^2^. For interpretation, a circularity value of 1 represents a perfect circle [12]. The en-face angiogram of each plexus was processed using the open-source toolbox OCTAVA [8] that calculates the VD, total vessel length (TVL), vessel length density (VLD), vessel diameter and tortuosity, branchpoint density (BD) and fractal dimension (FD). The optimal Frangi filter maximum kernel size was 4 pixels and the optimal twig size was 8 pixels as recommended [13]. A description of these parameters can be found elsewhere [8]. Figure 1 presents the graphical user interface of OCTAVA.

### Outcomes and sample size

The main outcome of this manuscript is the retinal VD. Based on a previous population-based study that assessed healthy subjects with OCTA [14] and the differences found so far in other studies enrolling SLE patients [15], the sample size was calculated for a difference of 5%, a power of 90%, and an alpha value of 0.05. Considering these parameters, a minimum of 14 subjects per group must be included. To account for the attrition rate and the need to exclude poor-quality images, we aimed to enroll 20 subjects per group.

The secondary outcomes include TVL, VLD, vessel diameter and tortuosity, BD and FD at both plexuses as well as FAZ parameters. These include area (mm^2^), perimeter (mm), and circularity, calculated using the following equation: (4π × area)/perimeter^2^.

### Statistical analysis

Continuous data is expressed as means and standard deviations (SD) or medians and interquartile ranges (IQR) according to its distribution. Categoric variables are described as absolute and relative frequencies. Parametric or nonparametric tests were used for continuous variables comparison between groups and correlation with disease duration for SLE patients and age for both groups, according to data distribution. For interpretation, correlation coefficients cut-offs were as defined by Chang [16]. Analyses were performed using STATA v14.2 (StataCorp LP, College Station, TX, USA).

## Results

Forty eyes of 40 caucasian women (20 SLE patients and 20 healthy subjects) with similar demographic and ophthalmological characteristics were assessed (Table 1). All SLE patients had early-onset disease with a mean ± SD duration of 7.35 ± 2.21 years. Table 2 details the clinical manifestations, laboratory features and treatments for SLE group. Most patients (65%) patients had no organ damage. Antiphospholipid antibodies, anti-double stranded DNA antibodies and low complement levels were present in 45%, 30% and 20% of patients, respectively. All patients were under HCQ and the mean ± SD cumulative and daily doses were 529.9 ± 200.5 g and 3.5 ± 0.8 mg/kg, respectively.

**Table 1:** Demographic and ophthalmologic characteristics of population

	SLE patients (*n* = 20)	Healthy controls (*n* = 20)	*P*-value
Age, years	36.9 ± 10.7	36.6 ± 9.4	0.527
Female sex	20 (100)	20 (100)	-
Ophthalmologic characteristics			
VA, logMAR	0.0 (0.0)	0.0 (0.0)	-
SE, D	0 [-2.0–0]	-0.4 [-2.5–0]	0.977
IOP, mmHg	13.9 ± 2.1	13.2 ± 1.5	0.887

Data shown as number (percentage), mean ± standard deviation and median [interquartile range]. D: diopters; IOP: intraocular pressure; SE: spherical equivalent; SLE: Systemic Lupus Erythematosus; VA: visual acuity

**Table 2:** Clinical SLE manifestations, laboratory features, and treatment of SLE patients

	SLE patients (*n* = 20)
Clinical features	
Mucocutaneous	16 (80)
Musculoskeletal	12 (60)
Serositis	2 (10)
Renal	0
Hematological	11 (55)
Neuropsychiatric	0
Antiphospholipid syndrome	6 (30)
Disease activity index	
SLEDAI-2 K, median	2.0 [0–2.0]
SLEDAI-2 K ≤ 4	20 (100)
Damage index	
SDI, median	0 [0–1.0]
SDI ≥ 1	7 (35)
Laboratory features	
ANA*	19 (95)
Anti-dsDNA*	6 (30)
Complement	
Low C3	1 (5)
Low C4	4 (20)
Antiphospholipid antibodies	
Lupus anticoagulant*	5 (25)
Anticardiolipin (IgG or IgM) *	7 (35)
Anti-β2-glycoprotein I antibodies (IgG or IgM) *	8 (40)
Triple positivity	5 (25)
Any positivity	9 (45)
Treatment	
Hydroxychloroquine	20 (100)
Glucocorticoids	5 (25)
Synthetic DMARDs	6 (30)
Biologic DMARDs	2 (10)
Immunoglobulins	1 (5)

Data are shown as number (percentage) and median [interquartile range]. ANA: antinuclear antibodies; Anti-dsDNA: anti-double-stranded DNA; DMARDs: disease-modifying antirheumatic drug; SDI: Systemic Lupus International Collaborating Clinics/American College of Rheumatology (SLICC/ACR) Damage Index; SLE: Systemic Lupus Erythematosus; SLEDAI-2 K: Systemic Lupus Erythematosus Disease Activity Index 2000. * The proportion of patients in each group with positive values for each autoantibody

**Fig. 2:**
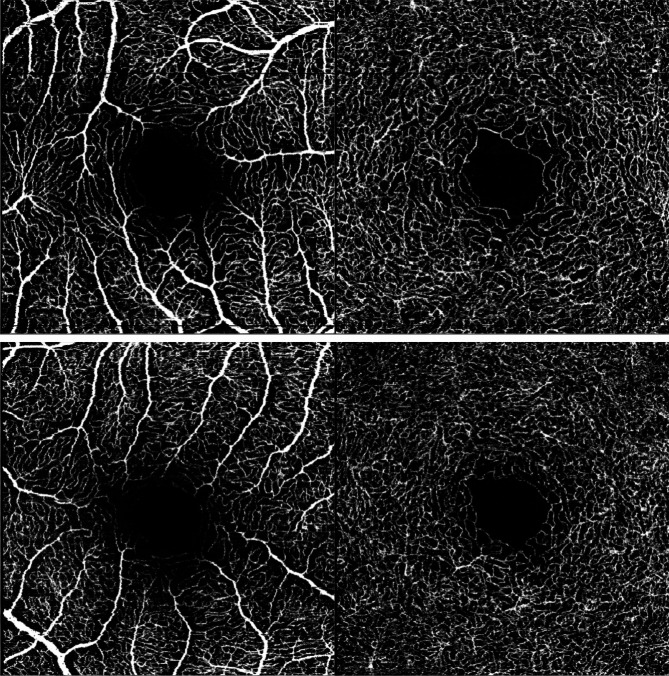
OCTA slabs obtained with Optical Coherence Tomography Angiography of a lupus patient (down) and an age-match healthy subject (up). Right and left slabs represent superficial and deep capillary plexuses (SCP and DCP), respectively. Lupus patient has a vessel density (VD) of 45% and 47% for SCP and DCP, respectively, whereas the healthy subject presents a VD of 57% and 56% for the same plexuses

The retinal vascular parameters are presented in Table 3; Fig. 2 presents representative OCTA images of an age-matched pair of lupus patient and healthy subject. No significant differences were found in FAZ parameters when comparing SLE and comparison groups. In the SCP, SLE group presented lower values for VD (*p* = 0.002), TVL (*p* = 0.027), mean diameter (*p* = 0.003), FD (*p* = 0.024), and tortuosity (*p* = 0.012). Similar findings were observed for DCP with SLE patients displaying lower values for VD (*p* = 0.002), TVL (*p* = 0.012), VLD (*p* = 0.017), mean diameter (*p* = 0.003), and FD (*p* = 0.022).

**Table 3:** Retinal vascular parameters

	SLE patients (*n* = 20)	Healthy controls (*n* = 20)	*P*-value
FAZ parameters			
area, mm^2^	0.376 ± 0.079	0.360 ± 0.108	0.601
perimeter, mm	2.208 ± 0.241	2.150 ± 0.320	0.518
circularity, mm^2^	2.301 ± 0.245	2.239 ± 0.350	0.519
Superficial capillary plexus			
VD, %	44.10 ± 12.72	57.55 ± 12.80	**0.002**
TVL, mm	73.31 ± 14.89	82.51 ± 9.88	**0.027**
VLD, %	3.15 ± 0.31	3.28 ± 0.198	0.130
Mean diameter, µm	27.65 ± 8.29	36.60 ± 9.61	**0.003**
BD, nodes/mm	4.57 ± 0.86	4.13 ± 0.50	0.057
FD	1.52 ± 0.03	1.54 ± 0.02	**0.024**
Tortuosity	0.14 ± 0.02	0.15 ± 0.02	**0.012**
Deep capillary plexus			
VD, %	46.50 ± 9.26	55.95 ± 8.68	**0.002**
TVL, mm	74.08 ± 13.87	84.66 ± 11.19	**0.012**
VLD, %	3.19 ± 0.23	3.36 ± 0.20	**0.017**
Mean diameter, µm	23.80 ± 4.87	28.90 ± 5.46	**0.003**
BD, nodes/mm	5.06 ± 0.74	4.76 ± 0.50	0.140
FD	1.55 ± 0.03	1.56 ± 0.02	**0.022**
Tortuosity	0.14 ± 0.02	0.15 ± 0.02	0.067

Data shown as mean ± standard deviation. *P*-values < 0.05 are displayed in bold. VD: vessel density; TVL: total vessel length; VLD: vessel length density; BD: branchpoint density; FD: fractal dimension. FD and tortuosity are dimensionless metrics

Considering the SLE group, a Pearson correlation coefficient was computed to assess the relationship between disease duration and the outcome variables. There was a moderate negative correlation between SLE duration and BD at SCP (*r* = -0.48, *p* = 0.030).

For both groups, the correlation between age and outcome variables was assessed with Pearson coefficient. FAZ parameters presented a moderate negative correlation with age (*r* = -0.43, *p* = 0.005 for area; *r* = -0.45, *p* = 0.005 for perimeter; *r* = -0.42, *p* = 0.007 for circularity). It was also found a negative weak correlation between age and TVL, VLD and FD at SCP (*r* = -0.31, *p* = 0.049 for TVL; *r* = -0.38, *p* = 0.017 for VLD; *r* = -0.34, *p* = 0.030 for FD).

## Discussion

SLE is a multiorgan autoimmune disease that may affect the eye with keratoconjunctivitis sicca being the most common ocular manifestation [6]. The visual morbidity is mainly attributed to retinal vascular and neuro-ophthalmological involvement. Subclinical ocular changes may arise before classic findings of eye involvement. OCTA provides a non-invasive assessment of retinal vasculature, allowing for early microvascular changes to be detected without the need of a dye [7]. In this study, we assessed the retinal vasculature of SLE patients without disease activity (SLEDAI-2 K ≤ 4) and no overt ocular manifestations and compared them with a demographically similar cohort of healthy subjects. Using OCTA, we found a rarefaction of the capillary network in superficial and deep capillary plexuses in SLE patients which was represented by lower vessel density, length, and diameter, as well as lower FD. To our knowledge, this is the first study applying this complete analysis of the retinal vascular network in adult patients with SLE.

Fundus fluorescein angiography (FFA) is the gold-standard for the assessment of retinal vasculature [17]. Tolda and colleagues [18] studied the eyes of active SLE patients with FFA and found several signs of microvascular involvement, with focal leakage, peripheral ischemia and attenuated tortuous vessels being the most frequent. Although most patients had clinical signs of retinopathy, the authors reported focal leakage, disc hypoperfusion and peripheral ischemia in patients with normal fundus examinations [18]. Despite being a reasonable option in patients with clinical signs of posterior segment involvement, FFA is not adequate nor cost-effective for patients without overt ocular manifestations since it is time-consuming, invasive and in most cases will not change the clinical decision. OCTA is a good option and is suited to routine clinical practice to study the macular vasculature of SLE patients without clinical signs of disease [7]. In patients with diabetic retinopathy, OCTA is a useful method to estimate peripheral retinal non-perfusion [19], a role that may also be valuable in other systemic diseases. Future research should evaluate the agreement between OCTA and FFA to determine the potential role of the former as a screening tool for selecting patients without overt changes that may benefit from FFA. The expected and warranted development of normative databases and standardized OCTA parameters may also positively influence its role in clinical practice.

In a previous work, we synthesized the evidence about the subclinical retinal microvascular changes in SLE as assessed by OCTA [7]. A significant reduction of macular VD in both plexuses in SLE patients was found when compared to healthy subjects but there were no significant differences in FAZ parameters. Of note, Tugan and coworkers [20] reported vascular impairment in the DCP but not SCP or FAZ parameters in patients with juvenile SLE, unveiling its potential to early detection of disease manifestations and confirming the higher susceptibility of this plexus to ischemia [21]. In this study, we did not find any differences in FAZ parameters, perhaps justified by the early nature of the disease and the considerable variation in the FAZ size, even among healthy subjects [22]. Bao and colleagues [23] found a decreased VD in SLE patients with lupus retinopathy versus those without retinopathy but did not study FAZ. Future research might explore the evolution of FAZ parameters in different phases of the disease.

In the present work, we aimed to characterize the retinal microvasculature in SLE beyond the quantification of VD, and we found that vessel density, length and diameter and FD were decreased compared to healthy subjects. In addition, SLE patients also presented less vessel tortuosity at SCP but not at DCP. Similar results were reported by Lee and colleagues [24], who quantitatively assessed FFA images of SLE patients and showed that retinal arteriolar narrowing was more frequent in this disease, and correlated these findings with inflammatory markers. Taken together, these results demonstrate a rarefaction of retinal vasculature in SLE, translated by vascular narrowing and shortening in the posterior pole. These findings are of clinical relevance as several studies recognized retinal microvascular signs as an early feature of atherosclerotic burden, associating retinal arteriolar and venular narrowing with the occurrence and development of hypertension [25, 26] and the risk of coronary heart disease and stroke mortality in middle-aged persons [27]. The association with hypertension is particularly relevant as this condition is highly prevalent among SLE patients [28] and a key risk factor for cardiovascular disease, and related to kidney damage [29]. From an ophthalmological point of view, this relation also deserves special consideration as lupus retinopathy resembles hypertensive retinal manifestations [30]. However, retinal microvascular involvement in SLE seems to result from immune complex-mediated microangiopathy rather than a hypertension-related consequence [31], as it might occur in the latter’s absence. The vascular changes herein found cannot be attributed to hypertension or other systemic diseases other than SLE as those were exclusion criteria. The rarefaction of central retinal microvascular network comes in line with the findings mentioned above of peripheral ischemia in SLE patients without overt ocular involvement [18]. As stated, OCTA might evolve to provide a surrogate of peripheral vasculature status [19] if certain conditions are met. Considering the association of retinal vascular findings and atherosclerotic burden, lupus end-organ damage and cardiovascular mortality, OCTA may also play a role in the prognosis and ascertainment of disease activity in the near future.

FD provides a unitless value that indicates the degree of complexity of a biological structure, enabling to assess a vasculature’s architecture and how completely it fills a specified area [32, 33]. In a two-dimensional space, a fractal pattern will have a FD between 1 and 2 with higher values demonstrating a more complex and denser vascular network [8]. A reduced retinal FD has been found in systemic diseases such as diabetes, hypertension and neurodegenerative disorders [32, 34, 35], representing the loss of smaller vessels. The reduced FD in SLE patients is in line with the remaining results, which point out to a subclinical attenuation of retinal microvasculature in this disease. In addition, this result supports the idea of angiopathy of small vessels in the pathophysiology of the disease. To our knowledge, this is the first demonstration of reduced FD in SLE. This parameter could be of use in OCTA image analysis software as it provides a unitless value that theoretically could be more reproducible. In fact, it has been shown to present good repeatability and reproducibility in healthy participants assessed by a swept-source OCTA [36].

SLE patients are especially vulnerable to retinal damage either by the disease or the treatments, as both can lead to visual impairment. The effect of SLE in retinal vasculature seems to be well established in active and inactive disease [7, 18] while the evidence linking the treatment with retinal vascular changes is not that robust. Although HCQ can lead to structural damage in the outer retina, a previous work from our group showed no evidence supporting its deleterious effect on retinal vasculature [37]. Some authors pointed to a protective effect, which is biologically more plausible [38]. Mimier-Janczak and colleagues [38] performed a sub-analysis to assess the potential influence of azathioprine and methotrexate on the retinal vascular network of SLE patients and found no significant differences [38]. While relevant, the direct effect of treatments in some settings might be difficult to tell from the disease process as the organization of comparable groups regarding a specific drug may not be ethical or reasonable.

This study has some limitations. A single investigator performed the measurement of FAZ parameters manually, as described. However, FAZ manual delineation was shown to have good repeatability and reproducibility in healthy subjects [39]. Images were analyzed by an external software, OCTAVA, as the device (Spectralis ^®^) used is still lacking an inbuilt software. This is one of the first studies resourcing OCTAVA, but the software provides metrics comparable to the literature and reproducible between devices [13]. In addition, OCTAVA allows a detailed description of the vascular network that can give invaluable clues to the pathophysiology of the changes observed. This study has several strengths. Patients were thoroughly selected and described, aiming to avoid confounder related to disease activity, treatment, or comorbidities. The reduced VD in SLE has already been established and well-documented in the literature, and it has been summarized in a systematic review by our group [7]. Hereby, we went further on the characterization of the microvascular changes and provided evidence that SLE reduces VD by affecting both diameter and length of microvasculature. Additionally, we used FD in this pathology for the first time and we believe that this unitless parameter can be more easily implemented across devices until a standardization of OCTA metrics is made available. In fact, it has been already used in different kinds of images, including with color fundus photography and FFA scans [34]. We studied the retinal vascular network in a non-invasive dye-free way, as used in routine clinical practice, and we shed light on the implications of the changes for local and systemic prognosis.

## Conclusion

In summary, SLE reduces retinal vessel density, diameter and length and FD without overt manifestations of the disease. These changes are consistent with SLE-associated microangiopathy and the peripheral ischemia found by others [18] in asymptomatic patients. Retinal vascular narrowing might have prognostic implications as it has been associated with higher cardiovascular burden and mortality [25–27]. Longitudinal studies are necessary to understand the role of these subclinical changes in the long-term evolution of these patients.

## Data Availability

The datasets used during the current study are available from the corresponding author on reasonable request.

## Abbreviations

ACR: American college of rheumatology
aPL: Antiphospholipid
CI: Confidence interval
CNS: Central nervous system
CVI: Choroidal vascularity index
DCP: Deep capillary plexus
EULAR: European league against rheumatism
FAZ: Foveal avascular zone
FD: Fractal dimension
FFA: Fundus fluorescein angiography
HCQ: Hydroxychloroquine
IQR: Interquartile range
MD: Mean diameter
OCT: Optical coherence tomography
OCTA: Optical coherence tomography angiography
OCTAVA: OCTA vascular analyzer
RA: Rheumatoid arthritis
RPE: Retinal pigment epithelium
SCP: Superficial capillary plexus
SD: Standard deviation
SD: OCT–spectral domain optical coherence tomography
SLE: Systemic lupus erythematosus
SLEDAI: 2 K–SLE disease activity index 2000
SS: Sjögren’s syndrome
TVL: Total vessel length
VD: Vessel density
VLD: Vessel length density
